# BDNF receptor TrkB as the mediator of the antidepressant drug action

**DOI:** 10.3389/fnmol.2022.1032224

**Published:** 2022-11-02

**Authors:** Plinio Casarotto, Juzoh Umemori, Eero Castrén

**Affiliations:** ^1^Neuroscience Center, HiLIFE, University of Helsinki, Helsinki, Finland; ^2^Gene and Cell Technology, A.I. Virtanen Institute, University of Eastern Finland, Kuopio, Finland

**Keywords:** antidepressant (AD), BDNF, TrkB, plasticity, parvalbumin interneurons

## Abstract

Brain-derived neurotrophic factor (BDNF) signaling through its receptor TrkB has for a long time been recognized as a critical mediator of the antidepressant drug action, but BDNF signaling has been considered to be activated indirectly through the action of typical and rapid-acting antidepressants through monoamine transporters and glutamate NMDA receptors, respectively. However, recent findings demonstrate that both typical and the fast-acting antidepressants directly bind to TrkB and thereby allosterically potentiate BDNF signaling, suggesting that TrkB is the direct target for antidepressant drugs. Increased TrkB signaling particularly in the parvalbumin-expressing interneurons orchestrates iPlasticity, a state of juvenile-like enhanced plasticity in the adult brain. iPlasticity sensitizes neuronal networks to environmental influences, enabling rewiring of networks miswired by adverse experiences. These findings have dramatically changed the position of TrkB in the antidepressant effects and they propose a new end-to-end model of the antidepressant drug action. This model emphasizes the enabling role of antidepressant treatment and the active participation of the patient in the process of recovery from mood disorders.

## Introduction

Soon after its discovery, it was recognized that the synthesis of brain-derived neurotrophic factor (BDNF) is regulated by neuronal activity (Zafra et al., 1990; Isackson et al., 1991; Dugich-Djordjevic et al., 1992). This finding laid foundation for the subsequent recognition of BDNF as the critical mediator of activity-dependent neuronal plasticity and connectivity during development as well as in the adult brain (Thoenen, 1995; Poo, 2001). As the limbic seizures used in early studies to induce BDNF mRNA expression in mice resembled seizures induced by the electroconvulsive shock therapy (ECT), these findings raised interest into a possibility that BDNF might be involved in the antidepressant mechanisms of ECT. Indeed, Ron Duman’s group found that ECT-like treatment in rats strongly increased the expression mRNA for BDNF as well as for its cognate receptor TrkB (neurotrophic tyrosine kinase receptor, NTRK2) in the hippocampus and cortex (Nibuya et al., 1995). Unexpectedly, they found that chronic treatment with antidepressant drugs also increase the expression of BDNF mRNA, albeit at lower level (Nibuya et al., 1995). Subsequent studies showed that BDNF injected into the midbrain region or hippocampus produces antidepressant-like effects in rodents (Siuciak et al., 1997; Shirayama et al., 2002). These pioneering findings led to the proposal of a central role of neurotrophic factors in the mechanisms of antidepressant drugs (Duman et al., 1997; Altar, 1999).

Another line of research that led to the recognition of the role of BDNF and TrkB in the mechanisms of antidepressant drug action is related to neuronal plasticity. It has been known for decades that the clinical effects of antidepressants appear after a delay of several weeks, although the biochemical effects of these drugs take place within minutes or hours. One potential explanation for this delay was that some kind of physical, time-consuming event might be required for the clinical effect to appear, and neuronal plasticity that involves physical growth and pruning was a natural candidate (Castrén, 2005). As BDNF signaling through TrkB is a key mediator of activity-dependent plasticity, BDNF was an excellent candidate involved in such a gradual growth process. Indeed, it was shown that antidepressant drugs reactivate a state of juvenile-like plasticity in the adult brain, a state that is called iPlasticity (Castrén, 2005; Umemori et al., 2018; Branchi and Giuliani, 2021). iPlasticity was first demonstrated as the reactivation of ocular dominance plasticity in the visual cortex (Maya Vetencourt et al., 2008), which is the classical model of developmental neuronal plasticity. Subsequent studies have demonstrated that antidepressants produce iPlasticity also in mood-relevant networks, such as the fear extinction and aggression control circuitries (Karpova et al., 2011; Mikics et al., 2018).

The finding that antidepressant treatments promote the proliferation and survival of newly-born neurons in the rodent hippocampal dentate gyrus further supported a role of long-lasting plastic changes in the antidepressant action (Malberg and Duman, 2003; Malberg et al., 2021). However, it later turned out that neuronal plasticity and BDNF signaling are also required for the rapid antidepressant effects of ketamine (Autry et al., 2011; Duman and Li, 2012; Liu et al., 2012), which undermined the role of plasticity as the explanation for the delay in the action of typical antidepressants. Indeed, this delay still remains a mystery. Together these two lines of research, activity-dependent BDNF regulation and its role in neuronal plasticity, laid foundation for the recognition of the critical role for BDNF-TrkB signaling in the mechanisms of antidepressant drug action (Duman et al., 1997; Nestler et al., 2002; Duman and Monteggia, 2006; Castrén and Monteggia, 2021).

## Brain-derived neurotrophic factor expression and signaling in the mechanism of antidepressant action

Essentially all antidepressant treatments tested so far have proven to increase the expression of BDNF mRNA and in most cases also BDNF protein levels. These treatments include typical antidepressants, including classical tricyclic, monoamine oxidase inhibitors, and serotonin-selective antidepressants (SSRI) (Nibuya et al., 1995; Duman et al., 1997; Altar, 1999; Russo-Neustadt et al., 1999; Coppell et al., 2003; Jacobsen and Mork, 2004; Duman and Monteggia, 2006; Calabrese et al., 2007, 2011), the rapid-acting antidepressants ketamine (Li et al., 2010; Autry et al., 2011; Autry and Monteggia, 2012; Lepack et al., 2014) and scopolamine (Wohleb et al., 2017), lithium (Jacobsen and Mork, 2004) as well as ECT (Nibuya et al., 1995; Jacobsen and Mork, 2004) and vagus nerve stimulation (Follesa et al., 2007; Biggio et al., 2009; Carreno and Frazer, 2014). Some authors have not found increases in BDNF with all antidepressants (Jacobsen and Mork, 2004), but doses, length of treatment and brain regions investigated may have contributed to this. BDNF mRNA levels are rapidly increase after ketamine and ECT (Nibuya et al., 1995; Autry et al., 2011), but several days of treatment with typical antidepressants are needed for the increase in BDNF mRNA and protein levels (Nibuya et al., 1995). The increase in BDNF mRNA levels by antidepressants may be produced by decrease in histone deacetylation at BDNF promoter regions (Russo-Neustadt et al., 2001; Dias et al., 2003; Tsankova et al., 2006; Karpova, 2014).

Antidepressants also promote BDNF release and signaling through TrkB (Castrén and Monteggia, 2021). Consistent with increased BDNF synthesis, typical as well as rapid-acting antidepressants increase TrkB autophosphorylation, which has been used as a proxy for BDNF release and binding to TrkB, and increase downstream signaling pathways activated by TrkB (Saarelainen et al., 2003; Rantamäki et al., 2007; Autry et al., 2011; Lepack et al., 2014). Antidepressants consistently increase the activation of phospholipase γ-1 (PLCγ-1) pathway (Saarelainen et al., 2003; Rantamäki et al., 2007), but the activation of extracellular signal regulated kinase (Erk)-pathway has also been reported to be activated (Duman et al., 2007; Lepack et al., 2016) and the activation of the Erk pathway may play a key role on the ketamine action (Li et al., 2010; Lepack et al., 2016).

The expression of BDNF mRNA and protein have also been investigated in humans with depression and antidepressant treatment. BDNF mRNA and/or protein levels have been reported to be reduced in postmortem brain samples of depressed patients (Dunham et al., 2009; Ray et al., 2011, 2014; Guilloux et al., 2012; Dwivedi, 2013) and suicide victims (Chen et al., 2001; Dwivedi et al., 2003, 2009; Pandey et al., 2008; Dwivedi, 2009; Youssef et al., 2018), and antidepressants restore the reduced levels (Chen et al., 2001). TrkB and phosphorylated TrkB have also been observed to be decreased in suicide victims (Dwivedi et al., 2003, 2009; Tripp et al., 2012). Similarly, serum BDNF levels are reduced in depressed patients (Karege et al., 2002) and successful antidepressant treatment normalizes these reduced levels (Shimizu et al., 2003; Gonul et al., 2005; Karege et al., 2005; Bocchio-Chiavetto et al., 2006, 2010; Yoshimura et al., 2007; Hellweg et al., 2008; Piccinni et al., 2008; Sen et al., 2008; Matrisciano et al., 2009; Molendijk et al., 2011, 2014; Rocha et al., 2016). However, as serum BDNF is derived from platelets (Yamamoto and Gurney, 1990; Radka et al., 1996; Fujimura et al., 2002; Lommatzsch et al., 2005; Naegelin et al., 2018), it is unclear to which extent serum BDNF levels correspond to brain levels (Seifert et al., 2010; Naegelin et al., 2018).

It has been widely considered that the effects of antidepressants on BDNF and TrkB signaling are indirect, mediated by the action of typical and fast-acting antidepressants on serotonin and NMDA-type glutamate receptors, respectively. However, recent findings have questioned the indirect action of antidepressants on BDNF and neuronal plasticity and revealed a direct binding of these drugs to TrkB (Casarotto et al., 2021).

## Antidepressants bind directly to TrkB

We recently discovered that essentially all antidepressant drugs directly bind to TrkB and thereby allosterically promote TrkB signaling (Casarotto et al., 2021). We first found that labeled fluoxetine and imipramine bind to TrkB and several orthogonal methods verified this binding. A point mutation in the TrkB transmembrane domain (TMD) (TrkB-Y433F) in the amino acids that are predicted to interact with antidepressants abolishes antidepressant binding to TrkB, indicating that fluoxetine binds directly to TrkB. Unexpectedly, we found that not only typical antidepressants, such as SSRIs and tricyclic antidepressants, but also the fast-acting antidepressant ketamine and its active metabolite R,R-hydroxynorketamine (R,R-HNK) (Zanos et al., 2016) directly bind to TrkB, and the effect of ketamine are also lost in the TrkB.Y433F mutants. This mutation as heterozygous abolishes the plasticity-promoting and antidepressant-related behavioral responses of both SSRIs and ketamine both *in vitro* and *in vivo*. It is important to note that such heterozygous mutation does not reduce BDNF binding to TrkB and heterozygous mice with this mutation do not show any behavioral phenotype (Biojone et al., 2022). Together these data suggest that direct binding to the TMD of TrkB is the common mechanism of action of both typical and fast-acting antidepressants.

**FIGURE 1:**
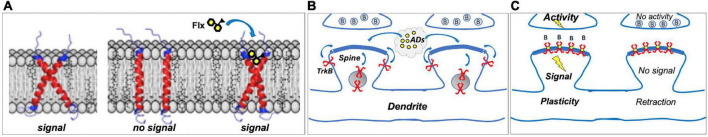
**(A)** Dimers of TrkB transmembrane domains (TMD) assume a crossed signaling-competent structure; in thick cholesterol-rich synaptic membranes, crossed structure becomes instable, and TrkB is excluded from synapses. Binding of fluoxetine (Flx) to the crossed TMDs acts as a wedge, stabilizing the signaling-competent structure in synaptic membranes. **(B)** Most TrkBs reside in vesicles or outside synapses; antidepressants (ADs) promote synaptic localization. **(C)** In Hebbian plasticity, active synapses are stabilized, whereas inactive ones are retracted, and brain-derived neurotrophic factor (BDNF) through TrkB is a critical mediator in this process. Antidepressants act as positive allosteric modulators of TrkB, promoting its localization in synaptic membranes where it can bind BDNF **(B)** released from stimulated synapses, enhancing TrkB signaling and stabilizing active synapses. Inactive synapses, however, do not release BDNF to activate synaptic TrkB receptors, which gradually leads to spine retraction. Direct TrkB agonist would activate TrkB in both active and inactive synapses, gradually leading to the decline in activity-dependent plasticity.

TrkB is a single TMD protein that is activated when the dimeric ligand BDNF induces the dimerization of two TrkB monomers, which leads to TrkB autophosphorylation and signaling. Atomistic simulations of TrkB TMD dimers revealed that TrkB TMD domains cross each other in the plasma membrane (Figure 1A). The positioning of the crisscrossed TrkB TMDs is determined by membrane thickness, which in turn is influenced by cholesterol concentrations. The crisscrossed conformation is stable in membranes with moderate cholesterol concentrations, supporting BDNF signaling, but in thick, cholesterol-rich membranes, such as synaptic membranes, the crisscrossed conformation of TrkB monomers tend to flip to parallel position (Figure 1A). TrkB in this parallel configuration does not appear to be stable and monomers are excluded from synaptic membranes (Suzuki et al., 2004; Pereira and Chao, 2007). Antidepressants bind to the outer crevice of the crossed transmembrane domains, interacting with both monomers. Antidepressant act as a kind of a wedge that stabilizes the crossed monomer configuration of the TrkB dimer, which increases the residence time of TrkB in the synapses, thereby enhancing the probability of BDNF binding and activation of TrkB (Casarotto et al., 2021; Figure 1A).

Essentially all the plasticity-related or antidepressant-like structural and behavioral responses to both fluoxetine and ketamine that we have tested so far are lost in heterozygous mice carrying the TrkB-Y433F mutation (Casarotto et al., 2021), which demonstrates that both of these antidepressants act by binding to the TrkB TMD. These responses include increased survival of newborn hippocampal neurons, promotion of ocular dominance plasticity, enhancement of object location memory, reduction of immobility in the forced swimming test and facilitation of fear extinction (Casarotto et al., 2021). It is important to note that these mice normally respond to BDNF and do not show any baseline behavioral deficits (Biojone et al., 2022), indicating that these behavioral effects are not mediated by any loss-of-function effects of BDNF responses, but are mediated by the inability of TrkB in these mice to bind antidepressants.

Although antidepressants bind to TrkB, they do not activate it on their own. Instead, they stabilize a configuration of TrkB dimers that promote the binding of BDNF, thereby allosterically potentiating the effects of BDNF onto TrkB (Figure 1). This is of physiological importance, since the effects of antidepressants as allosteric BDNF potentiators are confined to active synapses where BDNF is being released, whereas direct TrkB agonists would promote the stabilization of both active and inactive synapses. Therefore, antidepressant-potentiated BDNF signaling preserves and facilitates activity-dependent plasticity, which is a critical feature in both developmental and Hebbian plasticity (Thoenen, 1995; Park and Poo, 2013).

## Antidepressants and neuronal plasticity

The finding that antidepressants bind to TrkB links them directly to synaptic plasticity, but it has remained unclear how potentiated TrkB activity is translated into plasticity at a network level. Our recent work indicates that TrkB receptors specifically expressed on the parvalbumin (PV)-containing interneurons are critical in this regard. PV neurons have already been implicated in neuronal plasticity: their maturation coincides with the closure of critical periods of early life plasticity and inhibition mediated by PV neurons is reduced during iPlasticity induced by antidepressants or other treatments in the adult brain (Maya Vetencourt et al., 2008; Sale et al., 2010; Reh et al., 2020). We found that the antidepressant fluoxetine fails to induce iPlasticity in mice with reduced expression of TrkB in PV interneurons (Winkel et al., 2021). Conversely, activation of a light-sensitive TrkB (optoTrkB) specifically in the PV cells rapidly orchestrates a state of iPlasticity. Remarkably, optoTrkB activation replicated all the measures of iPlasticity induced by fluoxetine, including the reactivation of ocular dominance plasticity in the visual cortex (Winkel et al., 2021). The state induced by optoTrkB activation is characterized by a reduction in the excitability of PV cells produced by reduced expression and activity of Kv3.1 potassium channels and reduced output of inhibition to pyramidal neurons (Winkel et al., 2021). This leads to disinhibition of cortical pyramidal neurons and increased gamma oscillations, which in turn facilitates plasticity and underlies iPlasticity. It is remarkable that while activation of TrkB increases excitability of pyramidal neurons (Figurov et al., 1996), it reduces excitability of PV interneurons (Winkel et al., 2021), which is at least partially produced by the PV-cell specific expression of the Kv3.1 potassium channels. Therefore, activation of TrkB simultaneously in excitatory and inhibitory neurons do not counteract each other, but synergize, as the inhibition onto excitatory neurons is suppressed. It is important to note that as a consequence of TrkB activation, the PV interneurons are not simply shut down, which may result in uncontrolled excitability and seizures, but TrkB activity orchestrates a new state of PV cell activity that facilitates cortical plasticity in a controlled manner.

Antidepressants influence TrkB activity in PV neurons also through other mechanisms. We recently found that antidepressants disrupt the interaction between TrkB and the protein tyrosine phosphatase sigma (PTPσ) (Lesnikova et al., 2021). PTPσ interacts with TrkB and, when activated, restricts TrkB phosphorylation. Antidepressant-induced disruption of TrkB-PTPσ interaction therefore releases TrkB from this inhibitory control, promoting its activity. Interestingly, PTPσ is a receptor for chondroitin sulfate proteoglycans (Shen et al., 2009) that are the main constituents of perineuronal nets (PNN) that encase PV interneurons in the adult brain. It has been long known that disruption of PNNs by chondroitinase activates iPlasticity (Pizzorusso et al., 2002; Gogolla et al., 2009; Fawcett et al., 2019). PNN disruption is expected to reduce the activity of PTPσ and thereby facilitate TrkB activity. Indeed, we found that chondroitinase treatment fails to activate iPlasticity in mice with reduced expression of TrkB in PV interneurons (Lesnikova et al., 2021), demonstrating that TrkB activity in the PV cells is necessary for iPlasticity induced not only by antidepressants, but also by PNN disruption.

We have further found that antidepressant treatment also disrupts the interaction between TrkB and the adaptor protein complex-2 (AP-2) that is a critical mediator of endocytosis (Fred et al., 2019). Consequently, TrkB endocytosis is inhibited, which leads to increased plasma membrane localization of TrkB, thereby facilitating BDNF signaling.

Taken together, our recent findings show that antidepressants facilitate the ability of BDNF to activate TrkB receptors in PV interneurons through several distinct mechanisms: by directly binding to TrkB and allosterically increasing BDNF signaling (Casarotto et al., 2021); by inhibiting the dephosphorylation of TrkB through PTPσ; and by reducing TrkB endocytosis by disrupting the binding of AP-2 to TrkB. Together these mechanisms underlie the controlled disinhibition of pyramidal networks underlying iPlasticity and explain why TrkB in PV cells is particularly important for iPlasticity.

## Discussion

Previous studies have already established the critical role for BDNF-TrkB signaling in the antidepressant action (Castrén and Monteggia, 2021), but TrkB signaling has been seen as a secondary effect downstream of antidepressant binding to their various effector molecules, such as serotonin and noradrenaline transporters and NMDA receptors. New findings now propose a new end-to-end model of the antidepressant drug action. In this model, antidepressant drugs directly bind to TrkB receptors with low, but clinically meaningful affinity and thereby allosterically promote BDNF signaling in the plasma membranes of active synapses (Casarotto et al., 2021; Figure 1). Through intracellular signaling pathways downstream of TrkB, BDNF synthesis is increased and the translocation of AMPA-type glutamate at plasma membranes are increased. Activation of TrkB receptors particularly in PV-positive interneurons orchestrates a state of reduced activity of PV interneurons, which leads to disinhibition of pyramidal networks, turning on iPlasticity, a state of enhanced plasticity in the cortical networks (Winkel et al., 2021). iPlasticity sensitizes cortical networks to environmental experiences and facilitates rewiring of malfunctioning networks (Umemori et al., 2018; Branchi and Giuliani, 2021), leading to better adaptation to environment and mood recovery. Although a lot of research is needed for many details, this model provides a new framework for the understanding of the antidepressant action.

In the updated network model of depression, the initial event is binding of an antidepressant molecule to TrkB (Casarotto et al., 2021; Figure 1A). The finding that several different antidepressants, seemingly belonging to different chemical classes, such as SSRIs, tricyclic antidepressants, monoamine oxidase inhibitors, and also the rapid-acting antidepressants ketamine and R,R-HNK all bind to TrkB was unexpected. A recent finding failed to find interactions between R,R-HNK and TrkB or any other proteins (Bonaventura et al., 2022), however, concentrations of R,R-HNK tested may have been too low to detect binding to TrkB. Furthermore, in spite of promising preclinical findings (Hess et al., 2022) [but also see Shirayama and Hashimoto (2018)], whether R,R-HNK produces clinical antidepressant effects have been questioned (Farmer et al., 2020) and remain to be determined in clinical trials. If these findings are confirmed, they will overturn the dogma of the critical role of monoamines in the antidepressant drug action. However, it is clear that different antidepressants still bind to monoamine transporters and NMDA receptors and their contribution to the clinical outcome should become an active area of research. For example, the increased positive emotional bias seen early on during the SSRI treatment is likely mediated by serotonin and may play a significant role on the outcome (Harmer et al., 2004, 2017). With improved characterization of the binding site in TrkB, novel potential antidepressants with higher affinity to TrkB should be searched for.

One of the most unexpected aspects of the model of the critical role of TrkB binding in the antidepressant action is that a common binding site would mediate the effects of both fast and slow-acting antidepressants. It should be noted, however, that many different antidepressants reach higher than micromolar brain concentrations at the steady state, which is compatible with binding to TrkB (Renshaw et al., 1992; Karson et al., 1993; Bolo et al., 2000; Henry et al., 2000; Johnson et al., 2007). Intriguingly, it takes several weeks of continuous treatment to reach these micromolar fluoxetine concentrations (Karson et al., 1993). As the brain distribution of other SSRIs (Bolo et al., 2000; Henry et al., 2000) and also tricyclic antidepressants resemble that of fluoxetine (Daniel, 2003; Erb et al., 2016), these observations suggest the tantalizing hypothesis that gradual accumulation of antidepressants into brain at concentrations sufficient for binding to a low-affinity site, such as TrkB, may contribute to the slow onset (Kornhuber et al., 1995). In contrast, infusion of ketamine produces micromolar brain concentrations rapidly (Zanos et al., 2018), which is consistent with rapid onset of action. Therefore, although more research in this domain is needed, kinetic differences in the access of antidepressants to TrkB may be at least one factor influencing the delayed onset of action of typical antidepressants.

The action of antidepressants on network function helps to explain some discrepancies found in the behavioral responses to antidepressants. While administration of BDNF and TrkB agonists produce antidepressant-like effects on the cortex and hippocampus (Shirayama et al., 2002; Liu et al., 2010; Zhang et al., 2015a,b), TrkB antagonist ANA-12 paradoxically also produces antidepressant-like responses (Cazorla et al., 2011; Shirayama et al., 2015; Zhang et al., 2015a,b). Direct injection of ANA-12 to nucleus accumbens replicates the antidepressants effects, which is consistent with earlier studies showing that BDNF injection into this region produces a depression-like phenotype (Eisch et al., 2003). Therefore, TrkB activation does not produce antidepressant effects *per se*, but by enhancing plasticity promote the action of the particular network, which may ameliorate but also aggravate depression (Branchi and Giuliani, 2021).

The updated network hypothesis of antidepressant action is in many aspects very different from the traditional monoamine hypothesis. Although there is some evidence to suggest that BDNF signaling might be compromised in depression (Castrén and Monteggia, 2021), this model does not suggest that antidepressants act simply by restoring reduced BDNF signaling, but it emphasizes the role of BDNF-mediated plasticity that allows reorganization of networks through coherent activity provided by external and internal environment (Castrén, 2005; Branchi and Giuliani, 2021; Figures 1B,C). Such environmental activity can be enriched and guided by therapy or rehabilitation. However, although neuronal plasticity facilitates adaptation to changing environmental conditions, adaptation is not necessarily a positive phenomenon, but can become maladaptive if guides by an adverse environment (Alboni et al., 2017; Chiarotti et al., 2017; Branchi and Giuliani, 2021). Therefore, the model emphasizes that while antidepressants through facilitated plasticity enable recovery, they do not themselves cure depression, but active participation of the patient is required in the recovery process empowered by antidepressants.

## Author contributions

All authors listed have made a substantial, direct, and intellectual contribution to the work, and approved it for publication.
